# Detecting Succinylation sites from protein sequences using ensemble support vector machine

**DOI:** 10.1186/s12859-018-2249-4

**Published:** 2018-06-25

**Authors:** Qiao Ning, Xiaosa Zhao, Lingling Bao, Zhiqiang Ma, Xiaowei Zhao

**Affiliations:** 1 School of Information Science and Technology, Northeast Normal University, Changchun, 130117 China; 2 Key Laboratory of Intelligent Information Processing of Jilin Universities, Northeast Normal University, Changchun, 130117 China

**Keywords:** Predict succinylation sites, Multiple features, Grey pseudo amino acid composition, Information gain, SVM, Ensemble learning algorithm

## Abstract

**Background:**

Lysine succinylation is a new kind of post-translational modification which plays a key role in protein conformation regulation and cellular function control. To understand the mechanism of succinylation profoundly, it is necessary to identify succinylation sites in proteins accurately. However, traditional methods, experimental approaches, are labor-intensive and time-consuming. Computational prediction methods have been proposed recent years, and they are popular because of their convenience and high speed. In this study, we developed a new method to predict succinylation sites in protein combining multiple features, including amino acid composition, binary encoding, physicochemical property and grey pseudo amino acid composition, with a feature selection scheme (information gain). And then, it was trained using SVM (Support Vector Machine) and an ensemble learning algorithm.

**Results:**

The performance of this method was measured with an accuracy of 89.14% and a MCC (Matthew Correlation Coefficient) of 0.79 using 10-fold cross validation on training dataset and an accuracy of 84.5% and a MCC of 0.2 on independent dataset.

**Conclusions:**

The conclusions made from this study can help to understand more of the succinylation mechanism. These results suggest that our method was very promising for predicting succinylation sites. The source code and data of this paper are freely available athttps://github.com/ningq669/PSuccE.

**Electronic supplementary material:**

The online version of this article (10.1186/s12859-018-2249-4) contains supplementary material, which is available to authorized users.

## Background

As a type of widespread reversible post-translational modification, lysine succinylation plays a significant role in both eukaryotic and prokaryotic cells [1–3]. In succinylation procedure, the succinyl group (-CO-CH2-CH2-CO-) is covalent bonding to specific lysine residues in proteins which might lead to substantial chemistry changes to proteins [4]. Besides, lysine succinylation can induce mutations of charge in the environment with PH value (hydrogen ion concentration) range from − 1 to + 1 and promote structural and functional adjustment to substrate proteins [5]. It is extremely important to understand the molecular mechanism of succinylation in biological systems by identifying succinylated substrate proteins along with succinylation sites, so more and more focus is put on this field [6–23].

Many biological experimental methods have been developed to identify succinylated protein or succinylation sites, such as high performance liquid chromatography assays, spectrophotometric assays and liquid chromatography-mass spectrometry [24, 25]. However, these experimental approaches are inconvenient, time-consuming and costly, especially for large-scale data sets. Therefore, efficient computational prediction methods for the succinylated sites are urgently needed. Currently, numerous computational classifiers have been developed to identify PTM (Post Translation Modification) sites using various types of two-class machine learning algorithms [26–29]. We proposed a computational predictor, SucPred (2015), based on the combination of a kind of semi-supervised learning algorithm (Psol) and SVM classifier. This predictor took advantage of four types of sequence features, including autocorrelation function, encoding based on grouped weight, normalized van der Waals volume and position weight amino acids composition. Xu et al. (2015) built a predictor called iSuc-PseAAC based on SVM using Pseudo amino acid composition. And then, Xu et al. (2015) developed another predictor named SucFind. It was constructed based on SVM with k-spaced amino acid pairs and AAindex features. More recently, Hasan et al. (2016) proposed an approach SuccinSite based on Random Forest classifier. SucStruct predictor was built by Lopez et al. (2017) using structural properties of amino acids [30]. Thereafter, using profile bigram [31], PSSM-Suc [32] was introduced for identifying succinylation lysine sites by Lopez et al. (2017). Besides, they proposed Success predictor (2018) using evolutionary information of amino acids [33]. Thereafter, they (2018) used secondary structure information to further enhance the succinylation prediction [34]. Although these methods have already been developed to predict succinylation sites, there are some problems existing. First of all, the data set used in SucPred and iSuc-PseAAC was obtained from CPLM database [35] and the data set of SucFind was derived from several lysine modification databases and some relevant articles [36, 37], which are small and they didn’t cover novel succinylation data recently found. Besides, though the SuccinSite contains enough succinylation data, the performances of SuccinSite still have room for improvement.

**Fig. 1:**
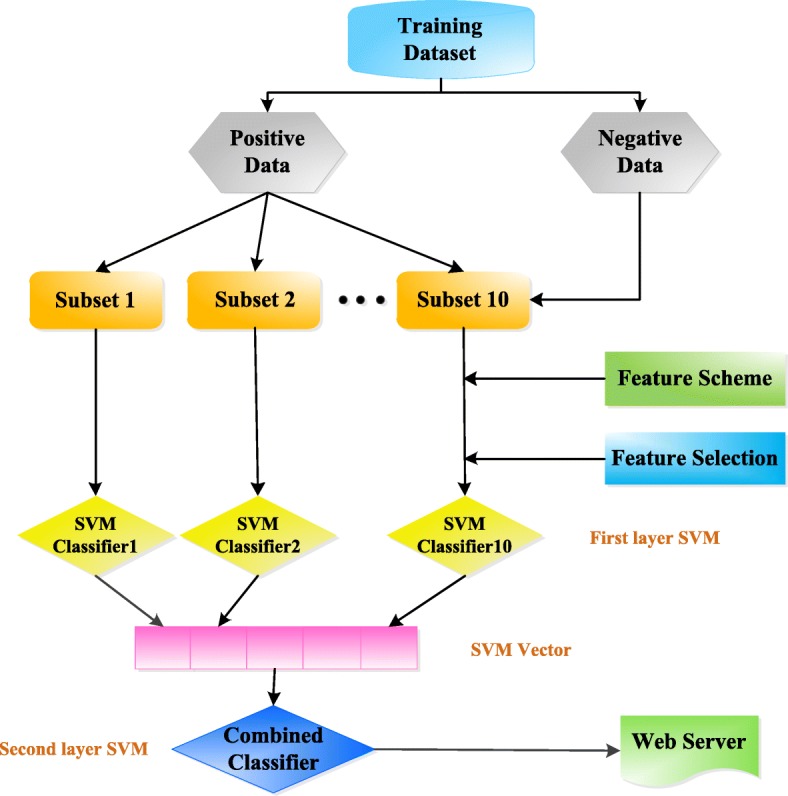
The flow chart of PSuccE

To solve problems mentioned above, we developed a new predictor, which was proposed to predict succinylation sites in protein using the same data set with SuccinSite. We used multiple efficient feature descriptors to derive informative features, including amino acid composition (AAC), binary encoding (BE), physicochemical property (PCP) and grey pseudo amino acid composition (GPAAC) and we showed the flow chart in Fig. 1. Finally, we obtained promising results with an accuracy of 89.14% and a MCC of 0.79 using 10-fold cross validation on training data set and an accuracy of 84.5%, a MCC of 0.2 on independent test set. These results demonstrated that this predictor is promising to predict lysine succinylation sites and could serve as a helpful tool to the community.

## Methods

As demonstrated in compliance with Chou’s 5-step rule [38] in a series of recent publications [6–12], we should follow the following five guidelines to establish a useful sequence-based predictor for a biological system: (a) select or construct a valid benchmark data set to train and test the predictor; (b) formulate these protein sequence samples with an effective mathematical expression that can truly reflect their intrinsic correlation with the target to be predicted; (c) introduce or develop a powerful algorithm to operate the prediction; (d) properly perform cross-validation tests to objectively evaluate the anticipated accuracy of the predictor; (e) establish a user-friendly web-server for the predictor that is accessible to the public. Below, we are going to describe how to deal with these steps one-by- one.

### Datasets

In this study, succinylation data was derived from UniProtKB/Swiss-Prot database and NCBI protein sequence database as Hasan et al. [29] did. After removing proteins that have more than 30% sequence identity to any other proteins in this dataset using CH-HIT, 2322 succinylation proteins including 5009 experimentally verified lysine succinylation sites were obtained. Then, 124 proteins were randomly separated from the 2322 proteins as an independent test set for testing, and the remaining proteins were training data set. We referred the experimentally verified lysine succinylation sites as positive sites, and all the lysine sites that lie on the same proteins as succinylation sites but don’t have any succinylation annotation were regarded as negative sites. Finally, 124 proteins with 254 succinylation sites and 2977 non-succinylation sites were obtained as independent test set, and 2198 proteins with 4755 succinylation sites and 50,565 non-succinylation sites as training set.

### Information entropy

Initially, we extract positive fragment and negative fragment utilizing the sliding window strategy, just like some other PTM site predictors [39, 40] The window size was set to L = 2 *l* + 1, where *l* is the number of upstream residues or downstream residues of the central amino acid (lysine). And ‘X’ was used when the number of flanking residues was less than *l*.

Nevertheless, not all the position within the window are contributing to the prediction of succinylation sites and even play a negative role. So it’s necessary to take measure to filter useful positions around the center lysine. The information gain is a measure of the amount of information [41]. The more orderly a system is, the lower the information entropy values, on the contrary, the more chaotic a system is, the higher the information entropy values. Therefore, information entropy is also a measure of the degree of ordering. Consequently, we utilized information entropy to select efficient position within the sliding window. Information entropy can be calculated as follows:

1 $$ {H}_c(x)=-{\sum}_{i=1}^n{p}_c\left({x}_i\right){\log}_2\left({p}_c\left({x}_i\right)\right) $$

where c represents the window size. x_i_ represents a kind of amino acid, and *n* = 20 denotes 20 kinds of different amino acid. p_c_(x_i_) means the probability that amino acid x_i_ appears at position c.

### General Pseudo amino acid composition

With the rapid growth of the amount of biological sequences in the post-genome era, one of the most significant but also most difficult problems in computational biology is how to convert a biological sequence into a numerical vector, yet still retain significant sequence-order information or key pattern characteristic, which is because almost all the existing machine-learning algorithms can only handle vector instead of sequence samples [22]. However, a vector that is defined in a discrete model may completely lose all the sequence-order information. To avoid this, the pseudo amino acid composition or PseAAC [42] was proposed. Ever since the concept of Chou’s PseAAC [43, 44] was put forward, it has penetrated into nearly all the areas of computational proteomics [45–50], many biomedicine and drug development areas [51]. Because of its widely and increasingly usage, two powerful open access soft-wares, named ‘propy’ [43] and ‘PseAAC-General’ [50], were released recently. In addition, a very powerful web-server called Pse-in-One [52] has been established and it can generate any desired feature vectors for protein/peptide and DNA/RNA sequences according to the need of users’ studies.

#### Amino acid composition

Amino acid composition feature is common and widely used in prediction of protein sequences (such as phosphorylation and acetylation and so on) [53, 54] as one kind of the most popular coding methods. AAC describes the frequencies of amino acids in protein sequences. In this work, AAC is the fraction of each type of amino acid in a sequence fragment. We calculated amino acid occurrence frequencies in the sequence surrounding the query site (the center site itself is not counted). There are 21 types of amino acids (including ‘X’) in total, thus 21 frequencies are calculated as features, the sum of which equal 1.

#### Binary encoding

The information of the type and position of the amino acid residues are basic but important to a protein sequence. Binary encoding scheme is the most intuitive method to acquire the positional characteristics of amino acids for protein sequences. It has been used in many kinds of PTM site prediction. If 20 amino acids are ranked as ACDEFGHIKLMNPQRSTVWY, it enciphered each kind of amino acid into a 20-dimension binary vector according to its position in this array. For example, A is replaced by 10,000,000,000,000,000,000, and Y is converted into 00000000000000000001. Especially, X is represent as 00000000000000000000.

#### Physicochemical property

AAindex is a database that includes numerical indices representing various physicochemical and biochemical properties of amino acids and pairs of amino acids [55]. Now it contains 544 PCPs in the version 9.0. An amino acid index is a set of 20 numerical values on behalf of various PCPs of amino acids. PCP has ever been successfully used in prediction of many protein modifications, such as S-glutathionylation and acetylation [56, 57]. In this work, we ranked these PCPs according to their abilities to distinguish between succinylation and non succinylation sites and used following top ten physicochemical properties: (1) consensus normalized hydrophobicity scale; (2) positive charge; (3) partition energy; (4) net charge; (5) conformational preference for all beta-strands; (6) conformational preference for antiparallel beta-strands; (7) mean polarity; (8) principal property value z3; (9) apparent partition energies calculated from Wertz-Scheraga index; (10) weights from the IFH scale.

#### Grey Pseudo amino acid composition

We combined Chou’s PseAAC [58, 59] and the grey model (GM (1,1)) [60] to convey protein fragments. It has already been successfully used in previous study [61–65]. GM (1,1) is an important and generally used approach in GM which can generate a series of regular data sequence by identifying difference between the trend of system factors, which also called correlation analysis. Assume that we have a known array

2 $$ {X}^{(0)}=\kern0.5em \left({x}^{(0)}(1),{x}^{(0)}(2),\dots, \kern0.5em {x}^{(0)}(n)\right) $$

which is irregular. Then, calculate the first-order accumulative generation operation (1-AGO) series for X^(0)^:

3 $$ {X}^{(1)}=\left({x}^{(1)}(1),{x}^{(1)}(2),\dots, {x}^{(1)}(n)\right) $$

in which x^(1)^(k) is computed by following equation:

4 $$ {x}^{(1)}(k)=\sum \limits_{i=1}^k{x}^{(0)}(i),\kern1em k=1,2,\dots, n $$

Next, an albinism differential equation can be gained according to X^(1)^:

5 $$ \frac{dX^{(1)}}{d(t)}+\alpha {X}^{(1)}=\beta $$

-α is the developing coefficient and -β is the influence coefficient. α and β are two elements of parameter vector *θ*.

6 $$ \theta ={\left[\alpha, \beta \right]}^T $$

*θ* can be calculated using a least square estimator.

7 $$ \theta ={\left[\alpha, \beta \right]}^T={\left[{B}^TB\right]}^{-1}{B}^TY $$

Where.

8 $$ B=\left[\begin{array}{cc}-0.5\left({x}^{(1)}(1)+{x}^{(1)}(2)\right)& 1\\ {}-0.5\left({x}^1(2)+{x}^{(1)}(3)\right)& 1\\ {}\dots & \dots \\ {}-0.5\left({x}^{(1)}\left(n-1\right)+{x}^{(1)}(n)\right)& 1\end{array}\right] $$

9 $$ Y=\left[\begin{array}{c}{x}^{(0)}(2)\\ {}{x}^{(0)}(3)\\ {}\dots \\ {}{x}^{(0)}(n)\end{array}\right] $$

In view of this, some important information are covered in coefficients. In this work, we incorporated PseAAC into these coefficients to reflect the difference between the positive data and negative data. The first arrays X^(0)^ were obtained from the physicochemical property which is described above. Each kind of AAindex corresponds to a series of X^(0)^ and works out a pair of coefficients.

Totally, we obtained 791 dimensions of features, including 21 dimensions for AAC (Amino Acid Composition), 500 dimensions for BE (Binary Encoding), 250 dimensions for PCP (Physicochemical Property) and 20 dimensions for GPAAC (Grey Pseudo Amino Acid Composition).

### Feature selection scheme

Not all features are equally important. Some features may not be relevant to the prediction of succinylation sites or they could be redundant with each other. Therefore, we performed a feature selection method IG (Information Gain) to remove the irrelevant and redundant features [66]. IG indicates the quantity of information a feature can bring to the classification system. The more information a feature brings, the more important it is. Thus the information gain can be utilized to evaluate the contribution of each feature to the classification. The formula of IG is as follows.

10 $$ IG(x)=E(x)-{\sum}_{v=1}^V\frac{\left|{x}^v\right|}{x}E\left({x}^v\right) $$

where x means a dimension of feature, and E(x) is the information entropy value of x. V means the amount of different values in each dimension feature x, and x^v^ (v = 1,2,...,V) indicates the probable value in feature x, and E(x^v^) is the corresponding information entropy value to x^v^.

### Ensemble learning

Ensemble Learning is one of the four main research directions in the field of machine learning. It uses multiple classifiers to solve the same problem, significantly improving the generalization ability of learning system. In our training data set, the amount of negative data (50565) is much larger than the amount of positive data (4755), so we adopted ensemble learning to resolve the unbalance between them.

We used Bootstrap Sampling to extract different subset data [67, 68]. It gets the difference of the base classifier through the difference of the training set. First, ten subsets with 4750 data were randomly selected from negative training data, and there is no coincidence between any two subsets. Then, combine every subset with the whole positive training data, respectively. Now, we have ten training data subsets with 9510 data, and we make a feature selection for each data subset using independent test set. After selecting the optimal feature group for every train data set, 10 SVM classifiers were obtained as the first layer classifiers. Next, we collected the results from the first layer classifiers and combined them as the feature of the second layer classifier. Finally, we predicted with the second layer classifier.

### Performance assessment

Independent test, subsampling test, and jackknife test are three commonly used cross validation methods to examine a predictor [69]. The jackknife test is deemed as the most reliable one among them [70]. However, n-fold cross validation test is commonly used instead of jackknife test because it can save much time. This method divides dataset into n equal subsets randomly, every n-1 of which are used for training and the rest one for testing. The procedure repeats several times and final result is calculated by averaging the accuracy of the n testing subsets. In this study, independent test and 10-fold cross validation were both used for evaluating the predictor.

Four measurements are generally used to evaluate the predictor: sensitivity (Sn), specificity (Sp), accuracy (Acc) and Mattew’s correlation coefficient (MCC). They are defined as follows:

11 $$ Sp=\frac{TN}{TN+ FP} $$

12 $$ Sn=\frac{TP}{TP+ FN} $$

13 $$ Acc=\frac{TP+ TN}{TP+ FP+ TN+ FN} $$

14 $$ MCC=\frac{TP\ast TN- FP\ast FN}{\sqrt{\left( TP+ FN\ast \left( TP+ FP\right)\ast \left( TN+ FN\right)\ast \left( TN+ FB\right)\right)}} $$

where TP, TN, FP and FN means the number of true positive, true negative, false positive and false negative, respectively.

This set of metrics is valid for the single-label systems instead multi-label systems. As for the multi-label systems, which exists frequently in system biology and system medicine [11, 71, 72], match with another completely diverse set of metrics as showed in [73].

## Result and discussion

### Optimal choice of positions

**Fig. 2:**
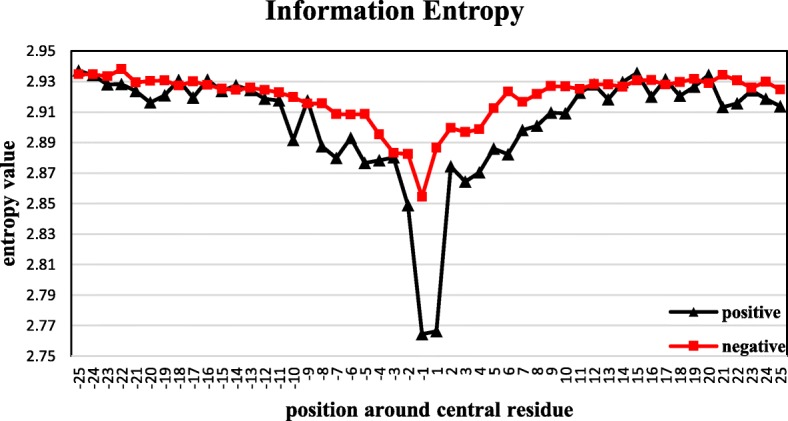
The information entropy value of positions around the central residue

In this study, we used information entropy (IE) to evaluate the importance of positions. Firstly, we chose 51 as the initial window size, with 25 amino acid residues upstream and 25 amino acid residues downstream. And then the entropy of each position was calculated by the formula (1). Entropy values are shown in Fig. 2.

**Fig. 3:**
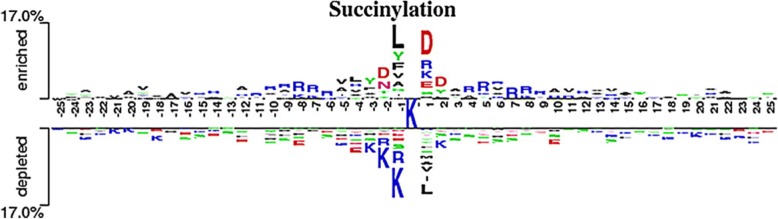
Two sample logos of the compositional biases around succinylation sites compared to non-succinylation sites. Statistically significant symbols are plotted using the size of the symbol that is proportional to the difference between the two samples. Residues are separated in two groups: (1) enriched in the positive samples; and (2) depleted in the positive samples. Color of the symbols was classified according to the polarity of the residue side chain

As we can see in Fig. 2, nearly all the information entropy values for positive data are lower than the values for negative data, which indicates that information entropy can be beneficial to distinguish succinylation sites and non-succinylation sites. The closer to the central residues, the lower the entropy values are, especially for the position 1 and − 1 which corresponds to the difference between succinylaiton and non-succinylation according to the two sample logo [74]. We can speculate from this appearance that succinylation may enhance the conservation of the target lysine and its surroundings which is consistent with Fig. 3. Eventually, we chose 25 positions which have greater difference between positive information entropy values and negative information entropy values, including − 20, − 17, − 10, − 8, − 7, − 6, − 5, − 4, − 2, − 1, 1, 2, 3, 4, 5, 6, 7, 8, 9, 10, 16, 21, 22, 24, 25.

### Analysis of optimal features

Not all positions and features are equally important in a protein. In this study, information gain was employed to acquire an optimal feature subset. For each subset, feature selection was processed respectively. Table 1 shows the final number of features in every training dataset, and the MCC curves of the succinylation prediction on ten training datasets for different dimensions of features are shown in Additional file 1: Figure S1.

**Table 1:** The number of features in every training dataset

Training Datasets	Number of features	ACC	BE	PCP	GPAAC
Subset1	186	14	38	123	11
Subset2	191	14	34	133	10
Subset3	158	13	27	107	11
Subset4	112	12	14	77	9
Subset5	194	14	38	131	11
Subset6	177	14	30	122	11
Subset7	194	14	37	134	9
Subset8	88	9	6	64	9
Subset9	66	7	5	45	9
Subset10	45	4	5	30	6
Common features	34	4	5	19	6

As we can see, after feature selection, the numbers of features for ten training datasets are different. It strongly proves that there is otherness between these ten training datasets even though they are separated from one negative dataset, and otherness is the requirement for using the ensemble learning. In spite of the difference, there are also many common features in ten feature vectors, including 4 AAC features, 5 BE features, 19 PCP features and 6 GPAAC features. We also evaluate the performance change between before feature selection and after feature selection for ten subsets (Additional file 1: Figure S2 and Table S1). As we can see in Additional file 1: Figure S2 and Table S1, the value of Sn, Sp, Acc and MCC are larger after feature selection, and the value of AUC (area below ROC curve) obviously increase.

### Comparison between ensemble learning and single SVMs

Ensemble learning train combinations of base models, which may be decision trees, neural networks, SVM, or others traditionally used in supervised learning. In this study, Bootstrap Sampling was used to extract different subset data. There are 50,565 negative sites and 4755 positive sites in our training dataset, nearly 10:1 for ratio of negative and positive data, so we randomly select 4755 data from negative data for ten times and there is no coincidence between any two subsets. Therefore, we have 10 separate training data subsets, which contains 4755 positive samples and 4755 negative samples, respectively (1:1 ratio of positive and negative data).

**Fig. 4:**
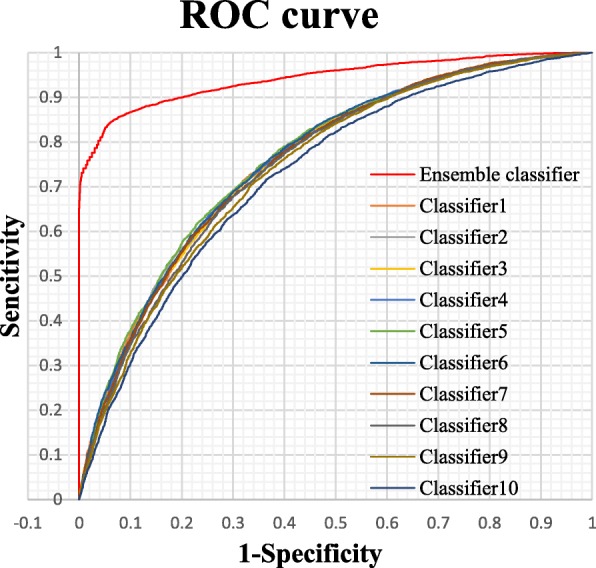
ROC curves (AUC) of predictions based on 10-fold cross validation

To verify if ensemble models perform consistently better than the single SVMs, we evaluate the performance of 10-fold cross validation on training dataset, and the results are shown in Table 2 and Fig. 4. As listed in Table 2, single SVMs always predict a lower Sp value and the Acc value are also not outstanding. After ensemble the training result from ten single SVMs, all the performances are obviously increased, especially for Sp, MCC and AUC.

**Table 2:** 10-fold cross validation performance of 10 subsets and ensemble classifier on training dataset

Training dataset	Sn (%)	Sp (%)	Acc	MCC
Subset1	72.29	66.91	0.6961	0.3926
Subset2	72.15	66.39	0.6927	0.3861
Subset3	72.21	66.33	0.6927	0.3861
Subset4	72.83	65.73	0.6929	0.3867
Subset5	71.69	67.24	0.6948	0.3898
Subset6	72.12	66.46	0.6930	0.3865
Subset7	71.94	65.64	0.6881	0.3767
Subset8	72.07	65.53	0.6880	0.3768
Subset9	72.97	63.52	0.6824	0.3665
Subset10	72.36	62.48	0.6742	0.3502
Ensemble	**84.31**	**93.97**	**0.89136**	**0.7864**

### Comparison between our method and existing methods

To further evaluate the performance of our method, we compared our method with four other existing predictors, SucPred, iSuc-PseAAC, SuccFind, SuccinSite and Success, using independent test dataset, including 254 succinylation sites and 2977 non-succinylation sites. Sn, Sp, Acc and MCC are used to measure the performance (Table 3). Because of the limitation of amount of independent test set, the result of independent test is not as good as 10-fold cross validation. However, when we control the threshold as 0.9 for these predictor, SucPred only obtain 67.3, 27.1% and 0.643 for Sp, Sn, and Acc, and the MCC value was only − 0.03. iSuc-PseAAC and Success have satisfying values of Sp, but the Sn and MCC values are lower. SuccFind and SuccinSite are favorable, while our method achieve a Sp of 88.6%, a Sn of 37.5%, an Acc of 84.5% and a MCC of 0.204, which were much better than SuccFind’s and SuccinSite’s performance. Because of the high value of threshold to guarantee the prediction of positive samples, the sensitivity values are less than the specificity value. The promising performance demonstrated that the this predictor was particularly useful for protein succinylation prediction.

**Table 3:** A comparison of PSuccE with existing predictors using an independent test set

Measurement*	SucPred	iSuc-PseAAC	SuccFind	SuccinSite	Success	PSuccE
Sp(%)	67.3	88.7	79.2	88.2	86.8	**88.6**
Sn(%)	27.2	12.2	25.2	37.1	14.2	**37.5**
Acc	0.643	0.827	0.750	0.842	0.811	**0.845**
MCC	−0.030	0.013	0.029	0.199	0.007	**0.204**

^*^ The threshold value was controlled as 0.9 for these predictors

## Conclusion

Here, we implement an application of Ensemble learning to protein succinylation prediction problem. Results show that our method is helpful to identification of succinylation sites. This work also indicated that Ensemble learning was a useful technique for combining weak classifiers and improving performance. We are looking forward that our method will give a powerful help for further studies of succinylation process.

## Additional file

Additional file 1: **Figure S1.** The MCC score of the optimal feature subsets. **Figure S2.** AUC (area below ROC curve) change between before feature selection and after feature selection for ten subsets. **Table S1.** The performance change between before feature selection and after feature selection for ten subsets. (DOCX 2693 kb)

## Abbreviations

AAC: Amino acid composition
Acc: Accuracy
BE: Binary encoding
FN: False negative
FP: False positive
GM: Grey model
GPAAC: Grey pseudo amino acid composition
IG: Information gain
MCC: Matthew correlation coefficient
PCP: Physicochemical property
PH value: Hydrogen ion concentration
PTM: Post translation modification
Sn: Sensitivity
Sp: Specificity
SVM: Support vector machine
TN: True negative
TP: True positive
