# Supplementary material for: Detecting Succinylation sites from protein sequences using ensemble support vector machine
Source: BMC Bioinformatics. 2018 Jun 25;19:237. doi: 10.1186/s12859-018-2249-4 (PMC6016146; doi:10.1186/s12859-018-2249-4)
Supplement: Supplementary file 1 — Figure S1. The MCC score of the optimal feature subsets. Figure S2. AUC (area below ROC curve) change between before feature selection and after feature selection for ten subsets. Table S1. The performance change between before feature selection and after feature selection for ten subsets. (DOCX 2693 kb) [file 12859_2018_2249_MOESM1_ESM.docx]

Figure 1. The MCC score of the optimal feature subsets

Figure 2. AUC (area below ROC curve) change between before feature selection and after feature selection for ten subsets.

Table 1. The performance change between before feature selection

and after feature selection for ten subsets.

| Training Datasets | Before feature selection | | | | After feature selection | | | |
| --- | --- | --- | --- | --- | --- | --- | --- | --- |
|  | Sn(%) | Sp(%) | Acc(%) | MCC | Sn | Sp | Acc | MCC |
| Subset1 | 70.87 | 61.87 | 0.6258 | 0.1794 | 72.29 | 66.91 | 0.6961 | 0.3926 |
| Subset2 | 71.26 | 61.30 | 0.6209 | 0.1780 | 72.15 | 66.39 | 0.6927 | 0.3861 |
| Subset3 | 71.65 | 62.65 | 0.6335 | 0.1884 | 72.21 | 66.33 | 0.6927 | 0.3861 |
| Subset4 | 70.87 | 60.97 | 0.6175 | 0.1739 | 72.83 | 65.73 | 0.6929 | 0.3867 |
| Subset5 | 72.05 | 62.28 | 0.6305 | 0.1882 | 71.69 | 67.24 | 0.6948 | 0.3898 |
| Subset6 | 71.65 | 60.93 | 0.6178 | 0.1779 | 72.12 | 66.46 | 0.6930 | 0.3865 |
| Subset7 | 71.26 | 61.81 | 0.6255 | 0.1811 | 71.94 | 65.64 | 0.6881 | 0.3767 |
| Subset8 | 71.65 | 62.41 | 0.6314 | 0.1869 | 72.07 | 65.53 | 0.6880 | 0.3768 |
| Subset9 | 69.69 | 61.47 | 0.6212 | 0.1705 | 72.97 | 63.52 | 0.6824 | 0.3665 |
| Subset10 | 70.47 | 61.57 | 0.6227 | 0.1754 | 72.36 | 62.48 | 0.6742 | 0.3502 |
